# In Situ TEM Imaging of Solution‐Phase Chemical Reactions Using 2D‐Heterostructure Mixing Cells

**DOI:** 10.1002/adma.202100668

**Published:** 2021-06-09

**Authors:** Daniel J. Kelly, Nick Clark, Mingwei Zhou, Denis Gebauer, Roman V. Gorbachev, Sarah J. Haigh

**Affiliations:** ^1^ Department of Materials and National Graphene Institute University of Manchester Manchester M13 9PL UK; ^2^ Department of Physics and Astronomy and National Graphene Institute University of Manchester Manchester M13 9PL UK; ^3^ Institute of Inorganic Chemistry Leibniz Universität Hannover Callinstr. 9 30167 Hannover Germany

**Keywords:** calcium carbonate, graphene liquid cells, heterostructure mixing cells, in situ transmission electron microscopy

## Abstract

Liquid‐phase transmission electron microscopy is used to study a wide range of chemical processes, where its unique combination of spatial and temporal resolution provides countless insights into nanoscale reaction dynamics. However, achieving sub‐nanometer resolution has proved difficult due to limitations in the current liquid cell designs. Here, a novel experimental platform for in situ mixing using a specially developed 2D heterostructure‐based liquid cell is presented. The technique facilitates in situ atomic resolution imaging and elemental analysis, with mixing achieved within the immediate viewing area via controllable nanofracture of an atomically thin separation membrane. This novel technique is used to investigate the time evolution of calcium carbonate synthesis, from the earliest stages of nanodroplet precursors to crystalline calcite in a single experiment. The observations provide the first direct visual confirmation of the recently developed liquid‐liquid phase separation theory, while the technological advancements open an avenue for many other studies of early stage solution‐phase reactions of great interest for both the exploration of fundamental science and developing applications.

## Graphical abstract

A novel 2D‐heterostructure mixing cell allows sub‐nanometer resolution characterization of calcium carbonate precipitation via nonclassical nucleation using scanning transmission electron microscopy. A trigger‐based mixing mechanism using controlled damage to the MoS_2_ membrane enables visualization of the entire reaction timeline with high spatial resolution, including liquid–liquid phase separation, formation of amorphous calcium carbonate, and crystallization.

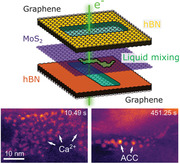

## 1 Introduction

Liquid‐phase transmission electron microscopy (LP‐TEM) is a powerful tool, which allows visualization of nanoscale processes in liquid environments, and has been broadly adopted by a wide range of disciplines including chemistry, materials science, geology, and the biosciences.^[^ ^1^, ^2^, ^3^ ^]^ The ability to study a specimen, either dispersed or dissolved in a liquid, while inside the TEM adds a dynamic perspective to this traditionally static characterization technique. Such imaging is accomplished by encapsulating a thin liquid layer between two electron transparent membranes, typically silicon nitride (SiN_x_) or graphene, to form a liquid cell (LC) ranging from tens of nanometers to several micrometers in thickness.^[^ ^4^, ^5^ ^]^ One of the most sought‐after aspects of LP‐TEM is the potential to conduct dynamic nanoscale studies of solution‐phase chemical processes and structural transformations.^[^ ^6^ ^]^ Among these, the synthesis of calcium carbonate is of particular interest due to its vital role in a great number of natural and industrial processes,^[^ ^7^, ^8^ ^]^ which remains an actively debated area due to the complex pathways through which a range of CaCO_3_ phases and crystalline polymorphs form.^[^ ^9^ ^]^

The benefits offered by LP‐TEM over conventional TEM and cryo‐TEM have already been used to garner new insights into calcium carbonate precipitation: Nielsen et al. have confirmed the coexistence of several nucleation pathways across multiple phases active in the precipitation of CaCO_3_^[^ ^10^ ^]^ and Smeets et al. have identified the binding of calcium ions in Ca‐polystyrene sulfonate globules as being a key stage in the formation of amorphous calcium carbonate (ACC) clusters.^[^ ^11^ ^]^ However, these studies used a commercial SiN_x_ LC design, which limits resolution to few nanometers and only allows mixing of the precursor solutions outside of the viewing area, meaning that the early stages of reaction cannot be captured.^[^ ^12^ ^]^ A trigger‐based method has been suggested by Stawski et al. to control the reaction timescale, however it requires a special reagent preparation which has limited application range, and the spatial resolution issue remains.^[^ ^13^ ^]^ Most recently, CaCO_3_ formation has been characterized using graphene liquid cells, achieving markedly higher resolution, though the lack of mixing capabilities means the electron beam was required to drive the reaction.^[^ ^14^ ^]^ To overcome these limitations, we have developed a 2D heterostructure mixing cell (2D‐MC) designed to provide both atomic resolution and controlled mixing of precursors in the immediate vicinity of the electron beam. We demonstrate the unique capabilities of 2D‐MC by studying the continuous evolution of calcium carbonate synthesis, though it should be noted that the general methodology can benefit studies of many other liquid–liquid mixing reactions.

**Figure 1:**
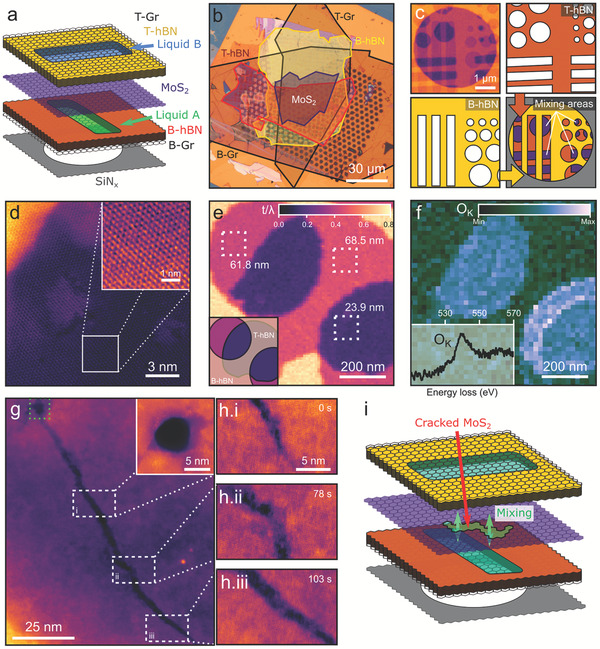
2D mixing cell design and working principle. a) Schematic of the graphene mixing cell before mixing. b) Optical microscopy image of a 2D‐MC prior to loading into the TEM showing individual flake layers. c) ADF‐STEM microscopy image of the 2D‐MC configuration (top, left) with overlays to show upper and lower hBN spacer layers (top right and bottom left, respectively). Mixing areas are colored purple in the combined legend (bottom right). d) Atomic‐resolution ADF‐STEM image of the MoS_2_ separation membrane suspended over a mixing area with an inset showing a high‐magnification view of the MoS_2_ lattice in this region. The bright region in the top left is the edge of the hBN spacer. e) EELS relative thickness map (*t*/λ) of a mixing area. f) EELS map of the oxygen K‐edge signal for the same area as (e) confirms aqueous solution in the upper wells. g) A beam induced pore in the MoS_2_ separation membrane (visible in the region highlighted with green dashed square and enlarged in the upper right inset) produces a crack emanating from the pore (115 nm long and 2–5 nm wide), which allows mixing of the upper and lower liquids. h) A time‐series shows kinking and splitting of the crack as it grows at 0.5 nm s^−1^ (full video in Video S1, Supporting Information). i) Schematic illustrating a 2D‐MC post‐mixing.

The base design for these 2D‐MCs consists of two adjacent liquid wells, separated by an atomically thin membrane that can be ruptured via local electron beam irradiation, causing liquid in the two pockets to mix. This is constructed by stacking two lithographically patterned hexagonal boron nitride (hBN) spacers,^[^ ^15^, ^16^ ^]^ few‐layer graphene upper and lower windows, and an inner MoS_2_ separation membrane, as shown in **Figure** **1**a (detailed fabrication procedure is described in Section S1 and Figure S1, Supporting Information). The optical image in Figure 1b shows a plan view of a typical 2D‐MC transferred onto a custom SiN_x_ grid with each 2D crystal shown in a different color to illustrate their alignment and coverage. Mixing areas occur where the wells in both hBN spacers overlap, such that the liquids are only separated by bilayer MoS_2_. This layout is illustrated in Figure 1c where the annular dark‐field (ADF) scanning transmission electron microscopy (STEM) image is accompanied by a layer schematic (conventional TEM of mixing area shown in Figure S2, Supporting Information). A high‐magnification ADF‐STEM image in Figure 1d shows the characteristic hexagonal atomic structure of the MoS_2_ membrane, with the yellow region in the top left corner corresponding to the hBN at the edge of the cell. Electron energy loss spectroscopy (EELS) was used to confirm the total thickness of the mixing regions (23.9 nm in Figure 1e) as well as the presence of water via detection of the oxygen K‐edge in the partially filled liquid wells (Figure 1f).^[^ ^15^, ^17^ ^]^ Further details of EELS including elemental analysis of precursors can be found in Section S3 and Figure S3 (Supporting Information).

## 2 Results and Discussion

To controllably instigate the mixing of the overlapping liquid pockets, a small area of MoS_2_ (5 × 5 nm^2^) is exposed to high electron fluence (6.8 × 10^6^ e^−^ Å^−2^), producing a pore in the separating membrane (inset in Figure 1g). Due to preexisting strain in the membrane, this causes a crack, typically several nanometers wide and a hundred nanometers long (Figure 1g), to propagate throughout the MoS_2_ membrane in the mixing region.^[^ ^18^ ^]^ A time series of the cracking process is shown in Figure 1h (Figures S4 and S5, full video in Video S1, Supporting Information). The crack allows local mixing of the two liquids (Figure 1i), a design that has several advantages over commercial flow‐based systems: 1) the earliest reaction stages can be studied as the mixing occurs near/at the imaging area; 2) each mixing event is confined to an individual pair of adjacent cells, allowing multiple experiments on a single grid; 3) mixing of precursors occurs over the length of the crack, including regions where the reaction is less affected by the electron irradiation. We find optimal performance is achieved when the graphene windows are 3–5 layer thick and the MoS_2_ is either mono‐ or bilayer such that a pore in the MoS_2_ can be created without compromising the integrity of the graphene outer windows. A 200 keV electron beam is preferred for the greater penetration, high probability of knock‐on damage to the MoS_2_, and improved resolution in the Titan ChemiSTEM used in these experiments.

While the 2D‐MC demonstrated in this work consists of graphene–hBN–MoS_2_–hBN–graphene, that is not to say this is the only configuration possible. The use of lithographically patterned hBN as a spacer results in a high‐quality seal between graphene and the atomically flat hBN, as well as between hBN and MoS_2_,^[^ ^19^ ^]^ ensuring there is no premature mixing. The use of MoS_2_ as the separation membrane for the 2D‐MC presented in this work is based on several factors: the ability to distinguish the separation membrane from other components of the cell using chemical mapping or high‐resolution imaging and the aforementioned cracking mechanism that has been characterized in other studies.^[^ ^18^ ^]^ However, we are optimistic that other 2D materials could be incorporated into the 2D‐MC as either separation membrane, spacer, or even window material, depending on experimental requirements.

**Figure 2:**
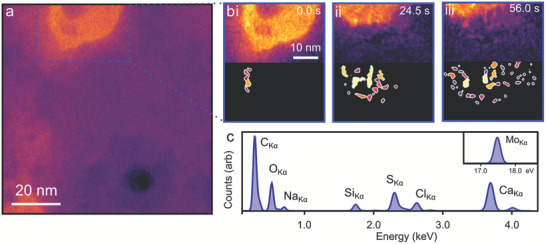
Phase separation after mixing observed in 2D‐MCs. a) After mixing, a bright (denser) liquid globule forms (top left of image) near a pore in the MoS_2_ membrane (bottom right). b) Time series of STEM‐ADF images (filtered to enhance contrast) showing the presence of smaller bright features as the globule contracts during imaging. An electron flux of 1.5 × 10^3^ e^−^ Å^−2^ s^−1^ was used. Corresponding segmented images that map changes in intensity are shown below (see the Supporting Information for detailed methods). c) Background fitted low energy part of the EDS obtained from the globule region after acquisition of the time series with high energy Mo_Kα_ peak included in the inset.

In order to use our new 2D‐MC to monitor the early stages of calcium carbonate precipitation in real time, the upper and lower liquid wells are filled with 7.5 × 10^−3^ m CaCl_2_ and 7.5 × 10^−3^ m Na_2_CO_3_ respectively. The solvent for both solutions is a 3:1 mixture of deionized water and isopropyl alcohol (IPA), with the latter included to promote cell filling and act as an inhibitor for beam‐induced reactions by scavenging molecular radicals.^[^ ^20^ ^]^ An ADF‐STEM image of a mixing area immediately after mixing is shown in **Figure** **2**a. Shortly after the mixing is triggered, we observe formations of irregularly shaped, high‐intensity liquid globules attached to the MoS_2_ membrane (top left of Figure 2a). Elemental analysis with energy‐dispersive X‐ray spectroscopy (EDS) shows such globules contain a high concentration of Ca, C, and O, as well as smaller amounts of Na and Cl (Figure 2c). In other videos (Video S2, Supporting Information) such brighter (denser) regions of liquid (30–150 nm diameter) can be seen fully suspended and hence moving across the field of view too quickly to be clearly imaged, occasionally picking up and dropping denser species attached to the membrane. Given the concentrations of the precursor solutions (see the Supporting Information for initial ionic activity product estimation), we propose these droplets to be evidence of post‐mixing spinodal decomposition in the liquid where calcium and carbonate ions have spontaneously phase‐separated to yield a dense liquid in a diffusion‐controlled manner, consistent with previous static studies.^[^ ^21^, ^22^ ^]^ Indeed, these observed globules are similar in morphology to the phase‐separated structures observed at early (<100 ms) reaction times using cryo‐TEM.^[^ ^23^ ^]^

**Figure 3:**
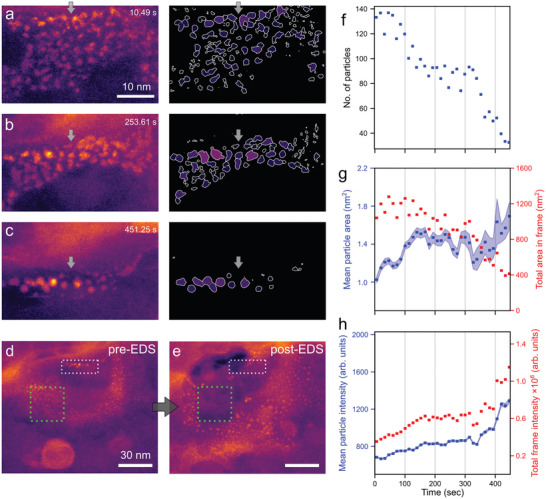
Visualization of calcium carbonate precipitation near to a crack created in the MoS_2_ membrane. a–c) ADF STEM images extracted from a time series showing particles forming by aggregation and coalescence of ion‐rich nanodroplets (segmented images highlighting the nanoparticles are shown to the right). The whole frame under went drift and background changes during the image series so as a reference the gray arrows have identical locations in (a)–(c). d) Wider field of view imaged immediately after the time series (a)–(c) with the scanned area of the time series shown by a dotted white rectangle. Evidently, many particles formed outside the scan area during the video acquisition. e) The same region after prolonged scanning (during EDS acquisition) of the region marked with a green dotted square. Again, particles have vanished in the scanned region but continued to form in the surrounding area. EDS analysis revealed high levels of Ca, C, and O. f–h) Various metrics (population, particle intensity and area, ensemble intensity and area) based on images in (a)–(c) showing their evolution over time. The mean values are indicated at 13.3 s time intervals (see the Supporting Information text and Figure S6, Supporting Information, for full details of image processing). An electron flux of 7.3 × 10^2^ e^−^ Å^−2^ s^−1^ was required to achieve adequate signal for accurate segmentation with sub‐nanometer resolution.

On further imaging, the dense liquid globules reduce in intensity and their perimeters recede to produce arrays of small, denser species with diameters of 1–3 nm (Figure 2b; Video S2, Supporting Information; and **Figure** **3**a–c). This process is likely a result of globule dehydration, with a high density of the small particles present in the imaging area, though similar small species are observed outside the field of view, in other areas not subjected to prolonged beam exposure. Based on these observations we propose that the effect of electron fluence is to accelerate an otherwise spontaneous dehydration process. This species appears to be amorphous, showing no crystalline order either in images or from selected area electron diffraction, while both measurements contained atomic detail from the MoS_2_ membrane (Figure 1d). This behavior is reproducible and observed for other globules within the pore vicinity (see Figure S6, Supporting Information, for image processing steps used to measure particle properties and Figure S7, Supporting Information, for further examples).

Statistical analysis of the video series (Video S3, Supporting Information) obtained during high‐resolution imaging of the region shown in Figure 3a–c and at a lower electron flux (7.3 × 10^2^ e^−^ Å^−2^ s^−1^) to prevent excessive dehydration, allows detailed understanding of the cluster evolution over the course of the experiment, with Figure 3d,e showing the reaction to have occurred over a much wider field of view. Segmentation and labeling of the species observed, detailed in Figure S6 (Supporting Information), allows the properties of the objects to be characterized over time. These measurements are presented in Figure 3f–h, where the frames have been binned along the time axis for clarity. Initially, a high number of increased intensity species with an average area of ≈1 nm^2^ (Figure 3a) are observed, each containing only a few (<10) hydrated ions, as per Figure 3f,g. These nanometer‐sized species are able to move across the substrate, facilitating multiple ripening and coalescence events, reducing the local population of such species from ≈140 to ≈100 during the first 180 s of imaging (Figure 3f). In the same period, the average area of the species in Figure 3g increases from 1.1 to 1.5 nm^2^, accompanied by a 14% increase in intensity (mass‐thickness contrast). Based on the frames in Figure 3a–c, growth by ripening tends to dominate in the first 60 s, after which coalescence becomes the predominant means of structural change. Between 180 and 320 s, the observed species become more spherical (Figure 3b), although their number and area remain roughly constant. Finally, from 320 to 500 s, frequent coalescence events dominate, reducing the population to 30 and their mean area to 1.7 nm^2^ (accompanied by a 50% increase in intensity) indicating either densification, 3D growth, or a combination. The small areas, irregular morphology, and low density of these species, shown quantitatively in the latter regimes of Figure 3f–h, suggest they are similar in character to prenucleation clusters (PNC),^[^ ^24^ ^]^ which are a key fixture of nonclassical nucleation theory.^[^ ^22^, ^25^ ^]^ The formation of calcium carbonate is a well‐known, but still debated, example of nonclassical nucleation, where rather than the direct monomer attachment that occurs in classical nucleation theory toward the formation of metastable nuclei, PNCs are stable, dynamic, chain‐like ionic associates. They form as direct precursors of phase‐separated nanodroplets that aggregate and dehydrate to an amorphous intermediate which subsequently restructures to form a crystalline product. For detailed context, we refer the reader to more comprehensive reviews of nonclassical nucleation theory, as it is too vast and complex a topic to consider fully in this text.^[^ ^25^, ^26^ ^]^ However, PNCs have been defined as being thermodynamically stable solute species with high structural and configurational dynamics that are precursors to binodal demixing events.^[^ ^25^ ^]^ Instead, we theorize the composition of the as‐formed dense liquid (post spinodal decomposition) equilibrates with the mother solution toward the denser composition given by the binodal curve. Thus, the splitting of the larger liquid globules into small denser nanodroplets reflects the equilibration of spinodal droplets toward the binodal composition, i.e., liquid–liquid coexistence where phase‐separated dense nanodroplets are in metastable equilibrium with the mother solution. Liquid–liquid separation has been proposed as a key step in the formation of amorphous calcium carbonate, where the PNC model considering thermodynamically stable ionic associates, rather than a fluctuating state of metastable ionic associates, has been shown to provide both explanatory and predictive power.^[^ ^22^ ^]^ The observed densification of the large, less dense (spinodal) liquid globules, transforming into smaller denser (binodal) droplets is likely kinetically facilitated by water transport, which could be enhanced by low surface tensions and the droplets being supported on the MoS_2_ surface. The final step, which was shown to occur over a much faster period, is the evolution of the droplets into ACC particles, which are much denser than the droplet precursors and have smooth, rounded morphologies.

A wider field of view is shown in Figure 3d, revealing that although species formation and densification is accelerated in the region scanned by the electron beam (white dotted rectangle), nanodroplets have formed within a much larger region, even at distances greater than 100 nm from the electron beam interaction volume. A similar phenomenon was observed while measuring EDS data (scanning in the green dotted square). Although intense irradiation during EDS analysis caused particle dissolution in the field of view (indicating the need to limit electron flux), many new droplets continued growing in the surrounding region. The morphological evolution of particles outside the imaging area is consistent with the imaged particles in Figure 3a–c (Video S3, Supporting Information) though as the process was not observed dynamically, we cannot be sure the mechanism is identical. There is evidence in the images of large globules, similar in morphology and image contrast to those in Figure 2, which move rapidly throughout the imaging area during Video S3 (Supporting Information) and which are static in Figure 3d,e. A lower magnification view of these globules is shown in Video S4 (Supporting Information), where they can be observed moving rapidly and changing morphology in a liquid‐like manner. As discussed earlier we consider these to be products of spinodal decomposition that occurs on mixing of the solutions as they are consistently seen in proximity of the initial product species (as in Figure S8, Supporting Information), only in this case a combination of changing surface energy, increased mixing time, and electron fluence causes detachment and motion of the globules themselves. Compared to ex situ studies of similar reactions using cryo‐TEM the size of both globules and nanodroplets reported here is at the lower end of the size distribution observed elsewhere,^[^ ^23^ ^]^ although similar size particles have been reported as subunits within larger amorphous nanoparticles.^[^ ^27^ ^]^ This difference is likely to be attributable to the improved imaging resolution in the 2D‐MC.

**Figure 4:**
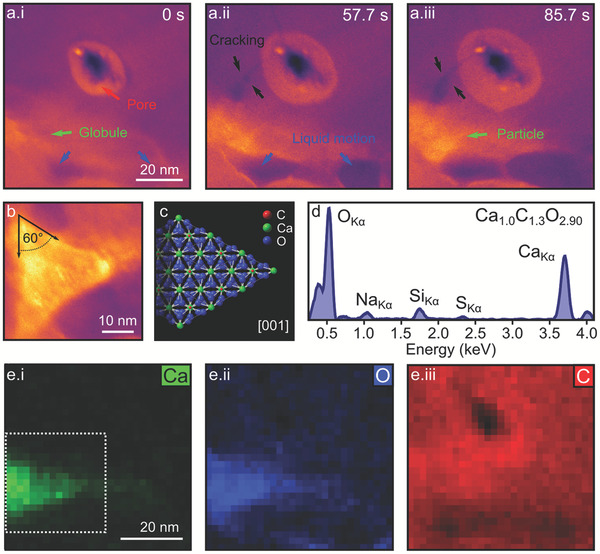
In situ crystallization of a calcite nanoparticle in a 2D‐MC. a) ADF‐STEM time series after mixing and prior to beam blanking. Both the beam induced pore and membrane cracking are visible (highlighted by red and black arrows, respectively). Liquid motion occurs near the hBN at the edge of the cell (blue arrows). The phase‐separated higher intensity liquid globule and initial particle formation are indicated by the green arrows. b) ADF‐STEM image taken after blanking the electron beam for 14 min. Image shows a faceted particle formed at the location of the globule in (a.i–a.iii). c) Crystallographic model for rhombohedral calcite viewed along the [001] direction (cleaved along center plane). d) EDS spectrum and quantitative composition acquired from particle area directly after formation. e) EDS elemental maps for Ca, C, and O of the region around the particle, with dashed white box showing region subject to quantitative EDS in (d).

Finally, we demonstrate the later stages of calcium carbonate precipitation where a crystalline phase of calcium carbonate is synthesized. To minimize potential electron beam‐induced artifacts we repeated the mixing procedure in a 2D‐MC, but blanked the beam after inducing the initial mixing (**Figure** **4**, where the beam induced pore created in the MoS_2_ membrane to induce cracking and mixing is visible in Figure 4a). Once mixing was established and the phase separation of Ca, C, and O‐rich, dense liquid globules became evident (green arrows), the electron beam is blanked for 14 min. On resuming imaging, the liquid globule has transformed to a triangular particle (Figure 4b), whose pronounced faceting indicates crystallinity. The 60° angles between facets suggest it is a rhombohedral calcite particle, which has nucleated via an amorphous (solid/liquid) precursor at the hBN edge (lower left corner of frame), causing growth only in one direction^[^ ^28^ ^]^ (Figure 4c), while quantitative EDS, acquired in situ from the 2D‐LC within minutes of crystal formation, confirms the composition to be CaCO_3_ (Figure 4d,e with background subtraction shown in Figure S9, Supporting Information). As with all LP‐TEM studies however, there is no way to completely eliminate the effects of the electron beam on the liquid, and confinement of such concentration‐dependent chemical processes into nanoscale volumes must also be accounted for when considering these results in the context of laboratory‐scale synthetic chemistry.^[^ ^29^ ^]^ However, the numerous similarities between the results presented here and those achieved using more traditional chemical analyses and reaction snap shots obtained via cryo‐TEM^[^ ^30^ ^]^ suggest that such effects can be minimized through experimental design to gain new insights into liquid mixing reactions.

## 3 Conclusion

Overall, the results presented here show that in situ mixing can be achieved directly in the field of view of a transmission electron microscope, with a subsecond time resolution and unrivalled, sub‐nanometer spatial resolution, through the use of 2D‐MCs. The unique combination of spatio‐temporal resolution and quantitative elemental analysis provided by the 2D‐MC can be used alongside other techniques to fill knowledge gaps in reaction timelines that were previously inaccessible. For example, the precipitation process observed here is consistent with in situ atomic force microscopy (AFM) investigations into the nucleation of organic molecules,^[^ ^31^ ^]^ but our in situ TEM 2D‐MC approach enables an order of magnitude improvement in both spatial resolution and time resolution. These enhanced parameters allowed us to characterize a dynamic multistage formation pathway that occurs as a result of mixing precursor solutions and consequently confirm the existence of liquid–liquid phase‐separation as well as reveal an indirect crystallization pathway of calcite, contrary to direct routes suggested in previous studies. We are confident this new methodology can be extended to many other mixing reactions by tailoring of the cell architecture (spacer thickness, cell shape, and dimensions) and composition (liquid concentrations, membrane material, window materials, seed crystals) to reveal exciting, new aspects of complex chemical processes.

## 4 Experimental Section

### 2D‐Heterostructure Mixing Cell Fabrication

The fabrication procedures used here to produce 2D‐MC are based on well‐documented procedures for 2D heterostructure fabrication^[^ ^32^ ^]^ and the steps are shown graphically in Figure S1 (Supporting Information). A mechanically exfoliated hBN flake of suitable flatness and thickness (30–60 nm) was coated with poly(methyl methacrylate) (PMMA) onto a silicon‐silicon oxide substrate and a mask created using electron beam lithography. Reactive ion etching transferred the lithographic pattern to the hBN, forming circular wells and trenches, followed by oxygen etching to functionalize the inner cell walls. The mask was then removed and the etched hBN flake annealed in H_2_/Ar. The patterned hBN flake was then transferred on top of a mechanically exfoliated few layer (3–5) graphene sheet to form the electron transparent window. To achieve this, the hBN was coated with PMMA, and KOH was used to dissolve the substrate and detach the sample. The PMMA‐supported flake hBN flake was then rinsed, dried, and finally transferred to the graphene using a micromanipulator.

A monolayer of MoS_2_ was then exfoliated mechanically onto a silicon–silicon oxide substrate and wet transferred onto the graphene–hBN stack as described above for the hBN, but now with a microdroplet of the required Liquid A pipetted between the MoS_2_ and the graphene–hBN stack as they are brought into contact. The sample, constituting the complete bottom half of the mixing cell, was then allowed to dry at room temperature for several hours.

The same process used to fabricate the bottom graphene–hBN stack was repeated to produce the top half of the 2D‐MC. This second graphene–hBN stack is similarly wet‐transferred hBN‐side down onto the MoS_2_ with a microdroplet of Liquid B pipetted between the layers. This is again left to dry over several hours before final transfer to a silicon nitride TEM support and a final cleaning step using organic solvents. For the majority of experiments presented in this work Liquid A and B are 7.5 × 10^−3^ m solutions of CaCl_2_ and Na_2_CO_3_ (Sigma‐Aldrich/Merck), with the solvent for each being a 3:1 mixture of deionized water and IPA, respectively.

### Characterization

Imaging in both TEM and STEM modes was performed using a probe‐corrected Titan G2 80‐200 S/TEM *ChemiSTEM* (Thermo Fisher Scientific) operated at 200 kV accelerating voltage. Annular dark‐field STEM was conducted with a probe current between 80 and 160 pA, dwell time of 2–20 µs, and a convergence semiangle of 21 mrad. Image processing (background subtraction, thresholding, segmentation) and corresponding statistical analysis was performed using numpy, scipy, and scikit‐image Python packages.

Electron energy loss spectroscopy was performed using a GIF Quantum ER System (Gatan Inc.) with an entrance aperture of 5 mm, 0.1 s total dwell time, and a dispersion of 0.25 eV/ch. Super‐X SDD EDS detectors (Thermo Fisher Scientific) were used for EDS spectrum imaging (collection solid angle of ≈0.7 s rad). Spurious Si counts were observed in EDS data due to characteristic X‐rays from the specimen inducing fluorescence in the Si‐based support and detector electronics (this effect is thus proportional to mass thickness). An uncertainty of ±20% was used in the standardless k‐factors used for quantification, resulting in an uncertainty of ≈5% for atomic percentage values. All spectroscopy analysis was conducted in the HyperSpy Python package.^[^ ^33^ ^]^

## Conflict of Interest

The authors declare no conflict of interest.

## Supporting information

Supporting Information

Supplemental Video 1

Supplemental Video 2

Supplemental Video 3

Supplemental Video 4

## Data Availability

All data sets and the applied image processing scripts are available from the authors on reasonable request.

## References

[1] F. M. Ross , Science 2015, 350, aaa9886. 26680204 10.1126/science.aaa9886

[2] J. J. De Yoreo , P. U. P. A. Gilbert , N. A. J. M. Sommerdijk , R. L. Penn , S. Whitelam , D. Joester , H. Zhang , J. D. Rimer , A. Navrotsky , J. F. Banfield , A. F. Wallace , F. M. Michel , F. C. Meldrum , H. Cölfen , P. M. Dove , Science 2015, 349, aaa6760. 26228157 10.1126/science.aaa6760

[3] H. Wu , H. Friedrich , J. P. Patterson , N. A. J. M. Sommerdijk , N. de Jonge , Adv. Mater. 2020, 32, 2001582. 10.1002/adma.202001582 32419161

[4] N. de Jonge , F. M. Ross , Nat. Nanotechnol. 2011, 6, 695. 22020120 10.1038/nnano.2011.161

[5] J. J. De Yoreo , N. A. J. M. Sommerdijk , Nat. Rev. Mater. 2016, 1, 16035.

[6] A. S. Kashin , V. P. Ananikov , Nat. Rev. Chem. 2019, 3, 624.

[7] B. Y. Shekunov , P. York , J. Cryst. Growth 2000, 211, 122.

[8] M. Faatz , F. Gröhn , G. Wegner , Adv. Mater. 2004, 16, 996.

[9] L. B. Gower , Chem. Rev. 2008, 108, 4551. 19006398 10.1021/cr800443h PMC3652400

[10] M. H. Nielsen , S. Aloni , J. J. De Yoreo , Science 2014, 345, 1158. 25190792 10.1126/science.1254051

[11] P. J. M. Smeets , K. R. Cho , R. G. E. Kempen , N. A. J. M. Sommerdijk , J. J. De Yoreo , Nat. Mater. 2015, 14, 394. 25622001 10.1038/nmat4193

[12] N. de Jonge , Ultramicroscopy 2018, 187, 113. 29428430 10.1016/j.ultramic.2018.01.007

[13] T. M. Stawski , T. Roncal‐Herrero , A. Fernandez‐Martinez , A. Matamoros‐Veloza , R. Kröger , L. G. Benning , Phys. Chem. Chem. Phys. 2018, 20, 13825. 29745416 10.1039/c8cp00540k

[14] K. S. Dae , J. H. Chang , K. Koo , J. Park , J. S. Kim , J. M. Yuk , ACS Omega 2020, 5, 14619. 32596599 10.1021/acsomega.0c01300 PMC7315570

[15] D. J. Kelly , M. Zhou , N. Clark , E. A. Lewis , A. M. Rakowski , M. J. Hamer , S. J. Haigh , R. V. Gorbachev , Nano Lett. 2018, 18, 1168. 29323499 10.1021/acs.nanolett.7b04713 PMC5821409

[16] A. Hutzler , T. Schmutzler , M. P. M. Jank , R. Branscheid , T. Unruh , E. Spiecker , L. Frey , Nano Lett. 2018, 18, 7222. 30346790 10.1021/acs.nanolett.8b03388

[17] N. Noh , J. Park , J. S. Park , K. Koo , J. Y. Park , J. M. Yuk , Lab Chip 2020, 20, 2796. 32633750 10.1039/d0lc00440e

[18] T. H. Ly , J. Zhao , M. O. Cichocka , L. J. Li , Y. H. Lee , Nat. Commun. 2017, 8, 14116. 28098140 10.1038/ncomms14116 PMC5253633

[19] M. J. Hamer , D. G. Hopkinson , N. Clark , M. Zhou , W. Wang , Y. Zou , D. J. Kelly , T. H. Bointon , S. J. Haigh , R. V. Gorbachev , Nano Lett. 2020, 20, 6582. 32786938 10.1021/acs.nanolett.0c02346

[20] T. J. Woehl , P. Abellan , J. Microsc. 2017, 265, 135. 27918613 10.1111/jmi.12508

[21] A. F. Wallace , L. O. Hedges , A. Fernandez‐Martinez , P. Raiteri , J. D. Gale , G. A. Waychunas , S. Whitelam , J. F. Banfield , J. J. De Yoreo , Science 2013, 341, 885. 23970697 10.1126/science.1230915

[22] J. T. Avaro , S. L. P. Wolf , K. Hauser , D. Gebauer , Angew. Chem., Int. Ed. 2020, 59, 6155. 10.1002/anie.201915350 PMC7187218 31943581

[23] J. Rieger , T. Frechen , G. Cox , W. Heckmann , C. Schmidt , J. Thieme , Faraday Discuss. 2007, 136, 265. 17955814 10.1039/b701450c

[24] D. Gebauer , A. Völkel , H. Cölfen , Science 2008, 322, 1819. 19095936 10.1126/science.1164271

[25] D. Gebauer , M. Kellermeier , J. D. Gale , L. Bergström , H. Cölfen , Chem. Soc. Rev. 2014, 43, 2348. 24457316 10.1039/c3cs60451a

[26] D. Gebauer , H. Cölfen , Nano Today 2011, 6, 564.

[27] Y. Xu , K. C. H. Tijssen , P. H. H. Bomans , A. Akiva , H. Friedrich , A. P. M. Kentgens , N. A. J. M. Sommerdijk , Nat. Commun. 2018, 9, 2582. 29968713 10.1038/s41467-018-05006-w PMC6030133

[28] Q. Hu , M. H. Nielsen , C. L. Freeman , L. M. Hamm , J. Tao , J. R. I. Lee , T. Y. J. Han , U. Becker , J. H. Harding , P. M. Dove , J. J. De Yoreo , Faraday Discuss. 2012, 159, 509.

[29] T. H. Moser , H. Mehta , C. Park , R. T. Kelly , T. Shokuhfar , J. E. Evans , Sci. Adv. 2018, 4, eaaq1202. 29725619 10.1126/sciadv.aaq1202 PMC5930397

[30] H. Su , B. L. Mehdi , J. P. Patterson , N. A. J. M. Sommerdijk , N. D. Browning , H. Friedrich , J. Phys. Chem. C 2019, 123, 25448.

[31] Y. Jiang , M. Kellermeier , D. Gebauer , Z. Lu , R. Rosenberg , A. Moise , M. Przybylski , H. Cölfen , Nat. Commun. 2017, 8, 15933. 28635962 10.1038/ncomms15933 PMC5482053

[32] A. V. Kretinin , Y. Cao , J. S. Tu , G. L. Yu , R. Jalil , K. S. Novoselov , S. J. Haigh , A. Gholinia , A. Mishchenko , M. Lozada , T. Georgiou , C. R. Woods , F. Withers , P. Blake , G. Eda , A. Wirsig , C. Hucho , K. Watanabe , T. Taniguchi , A. K. Geim , R. V. Gorbachev , Nano Lett. 2014, 14, 3270. 24844319 10.1021/nl5006542

[33] F. de la Peña , T. Ostasevicius , V. T. Fauske , P. Burdet , P. Jokubauskas , M. Nord , E. Prestat , M. Sarahan , K. E. MacArthur , D. N. Johnstone , J. Taillon , J. Caron , T. Furnival , A. Eljarrat , S. Mazzucco , V. Migunov , T. Aarholt , M. Walls , F. Winkler , B. Martineau , G. Donval , E. R. Hoglund , I. Alxneit , I. Hjorth , L. F. Zagonel , A. Garmannslund , C. Gohlke , I. Iyengar , H.‐W. Chang , hyperspy/hyperspy: Release v1.5.2, 2019, 10.5281/zenodo.592838 (accessed: January 2021).

