# Supplementary material for: In Situ TEM Imaging of Solution‐Phase Chemical Reactions Using 2D‐Heterostructure Mixing Cells
Source: Adv Mater. 2021 Jun 9;33(29):2100668. doi: 10.1002/adma.202100668 (PMC11469232; doi:10.1002/adma.202100668)
Supplement: Supplementary file 1 — Supporting Information [file ADMA-33-2100668-s002.pdf]

© 2021 Wiley-VCH GmbH

ADVANCED MATERIALS

Supporting Information

for *Adv. Mater.,* DOI: 10.1002/adma.202100668

In Situ TEM Imaging of Solution-Phase Chemical Reactions Using 2D-Heterostructure Mixing Cells

Daniel J. Kelly, Nick Clark, Mingwei Zhou, Denis Gebauer, Roman V. Gorbachev, and Sarah J. Haigh**

Supporting Information

In situ TEM imaging of solution-phase chemical reactions using 2D-heterostructure mixing cells

Daniel J. Kelly[1,3], Nick Clark[1,3], Mingwei Zhou[2,3], Denis Gebauer[4], Roman V. Gorbachev[2,3] and Sarah J. Haigh[1,3]**

Section 1: 2D-heterostructrure Mixing Cell Fabrication

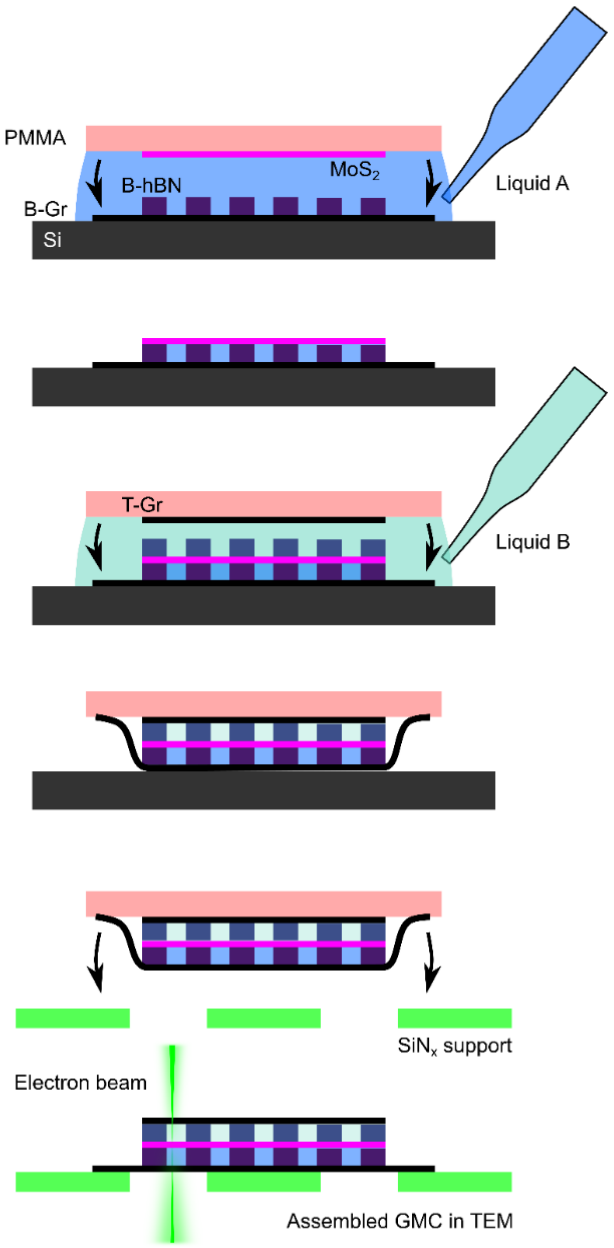

Figure S1.

Step-by-step fabrication schematic for 2D heterostructure mixing cells containing two solutions: Liquid A and Liquid B. Cell is mounted on an etched SiN_x support grid. Purple and

navy represent the bottom and top hBN flakes respectively, black lines are the graphene flakes, magenta is the MoS_2, blush pink is the PMMA support and green is the SiN TEM grid. Note that the in plane locations of upper and bottom wells do not perfectly match, as illustrated in main text Figure 1 and Figure S2a.

Section 2: Transmission Electron Microscopy

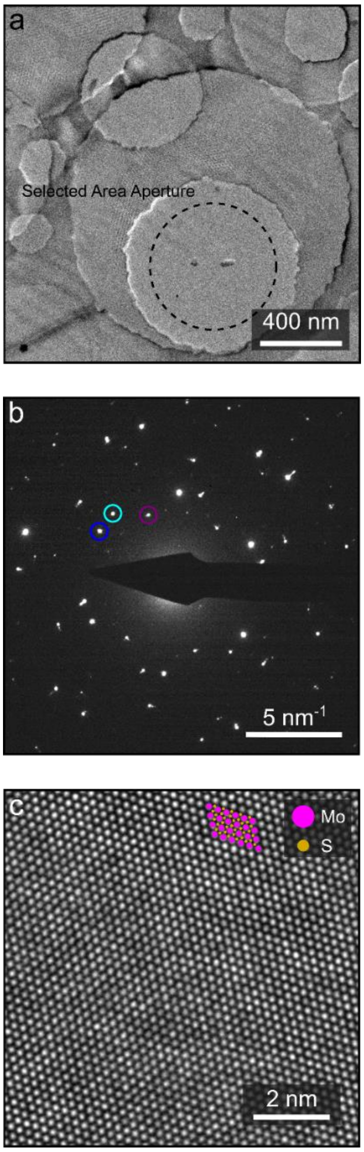

Figure S2.

a) Transmission electron microscope (TEM) micrograph of 2D-MC where overlaps in the arrays patterned in each hBN layer corresponds to a mixing region in which the stacking order is: graphene, liquid, MoS_2, liquid, graphene. b) A selected area electron diffraction pattern acquired at the mixing area (aperture position marked with a dashed circle in (a)) where distinct reflections are visible for both graphene layers (blue and cyan circles) and for the MoS_2 membrane (purple). c) Atomic resolution TEM shows the bilayer MoS_2 lattice with relatively few defects or contamination.

Section 3: Electron Energy Loss Spectroscopy

Electron energy loss spectroscopy (EELS) was used to assess the thickness of the different areas within a 2D mixing cell (2D-MC). as shown in Figure S3a, with the corresponding annular dark field scanning transmission electron microscope (ADF-STEM) micrograph in Figure S3c. Wells within bottom hBN (B-hBN) are not entirely circular, with a notable protrusion due to lithographic defect. Thickness measurements were made using the log-ratio method, where we consider four distinct regions each marked by a square in Figure S3a: hBN-hBN, liquid A-hBN, (here water-hBN), hBN-liquid B (here hBN-HMDS) and liquid A-liquid B (here water-HMDS). All regions contain the MoS_2 membrane as well as the top and bottom graphene sheets with a combined thickness of ~ 1-2 nm. Integrating over these areas and computing the inelastic mean free path for electrons at 200 kV (λ), gives absolute thickness values, annotated on the line profile in Figure S3a (lower panel). These were evaluated based on the effective atomic number method[1] which has previously been used in liquid-phase (LP)-EELS and shown to be a better estimation of thickness than Kramers-Kronig sum rule.[2] Even so it is important to note that these measurements have an error on the order of 10-15% and may underestimate λ.[3]

We consider the 2D-MC dimensions to be approximately symmetrical either side of the MoS_2 layer as both hBN spacers were deliberately chosen to be ~50 nm based on optical contrast, and the largest thickness value measured, t = 103.7 nm, is therefore in line with our expectations. Based on the values for water-hBN, hBN-HMDS, and water-HMDS (61.8 nm, 68.5 nm and 23.9 nm), we do not observe bowing of the graphene as is generally found in conventional graphene liquid cells **(**GLCs) without a spacer.[4] Instead the measurements suggest inverse bowing of the graphene that has been confirmed for spacer-based GLCs.[5] A cross-sectional illustration in the lower panel of Figure S3a, scaled according to the measured

thickness, illustrates this phenomenon, which likely arises from the perfect hermetic seal between the hBN and graphene and incomplete liquid filling. The high spatial resolution available in 2D-MCs can be partially attributed to this effect which is only possible due to the high tensile strength of graphene.[6] This results in extremely thin liquid layers, in this case ~15 nm per liquid compartment, though measurements made in other 2D-MCs show hydrated cells within thicker hBN can form liquid layers with thicknesses of up to 30 nm.

In order to further confirm hydration of the cells, energy dispersive X-ray spectroscopy (STEM-EDS) was used to map the oxygen K x-ray signal in Figure S3d, revealing an increase in the lower left mixing area, as well as a particularly high O concentration at the edge of the well in the upper right of the field of view. In addition, the oxygen *K*-edge signal characteristic of the water was detected using individual component analysis (ICA), (employing non-negative matrix factorisation) in Figure S3d and mapped in S3e, again showing a notable increase at the inner edge of the top right cell. This increased edge-concentration reflects the inverse bowing where only a thin liquid layer (10-30 nm) is maintained at the centre. The water is, as expected, present throughout the mixing area, but it is not detected where the water layer overlaps with the hBN spacer, likely as a result of plural scattering by the hBN reducing the signal to noise ratio of the relatively weak O K-edge x-ray signal.

This ring filling effect is also observed for some trench-shaped mixing cells, with one from a 2D-MC used to study the precipitation of $CaCO_3$ shown in Figure S4. Individual component analysis of correlated STEM-EDS and EELS data indicates higher concentrations of Ca, Cl and O near the trench walls. However, there is still a thin layer $CaCl_2$ (aq) in the centre of the mixing area. The quality of the detected core-loss EELS signal is also poorer for thin liquid layers in areas above or below the thick hBN spacer layers, due to loss of signal as electrons undergo plural scattering. This is likewise observed for STEM-EDS of the same sample, with ICA again revealing co-localisation of Ca, Cl and O. In addition, the observed widespread

oxygen presence throughout the trench implies a good degree of hydration within the trench.[7]

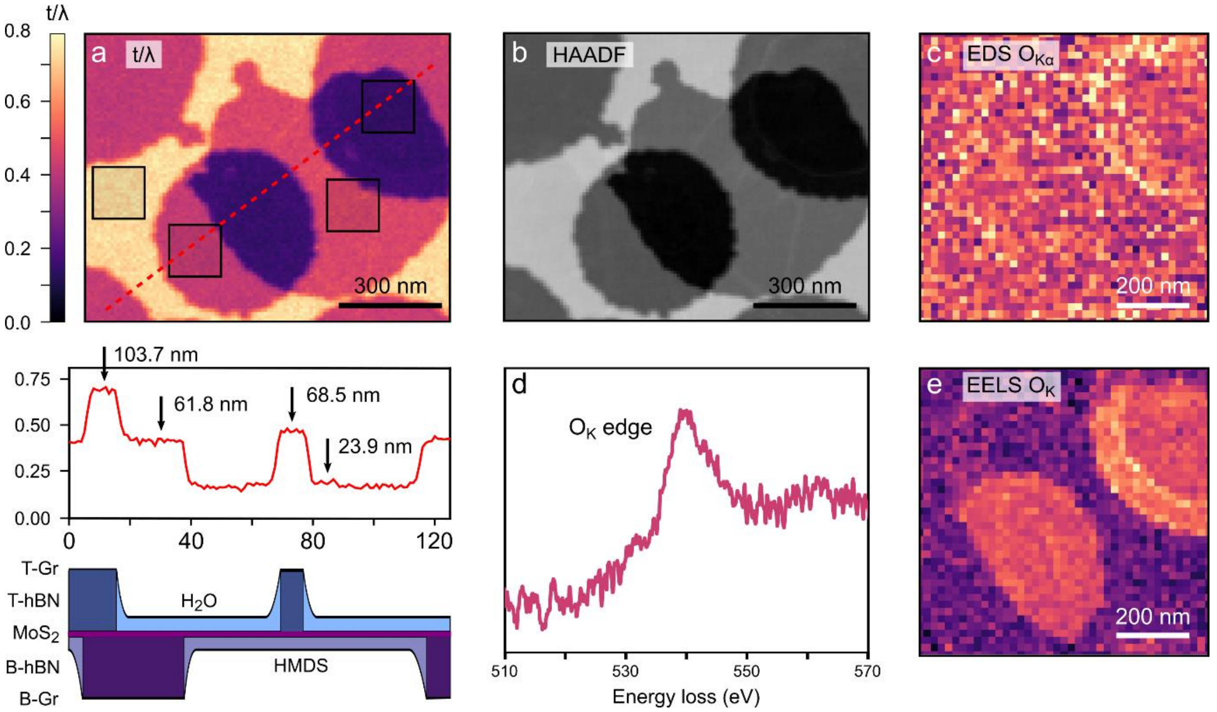

Figure S3.

a) Log-ratio thickness (t/λ) map of various areas in the 2D-MC. The lower panel shows absolute thickness values computed for each area marked by a black square, with an illustration of the cross section along the red dotted line shown to scale in the lower panel. **b**) Corresponding ADF STEM image of the area in **a**. **c**) Mapping the oxygen-containing EDS component with hotspots near the mixing area boundary. Signal-to-noise is low due to the low number of x-ray counts relative to EELS for the given acquisition time and light element species. **d**) The oxygen *K*-edge characteristic of water detected using individual component analysis. **e**) The oxygen-containing EELS component mapped over the area in **a**, showing hydration of the mixing cells.

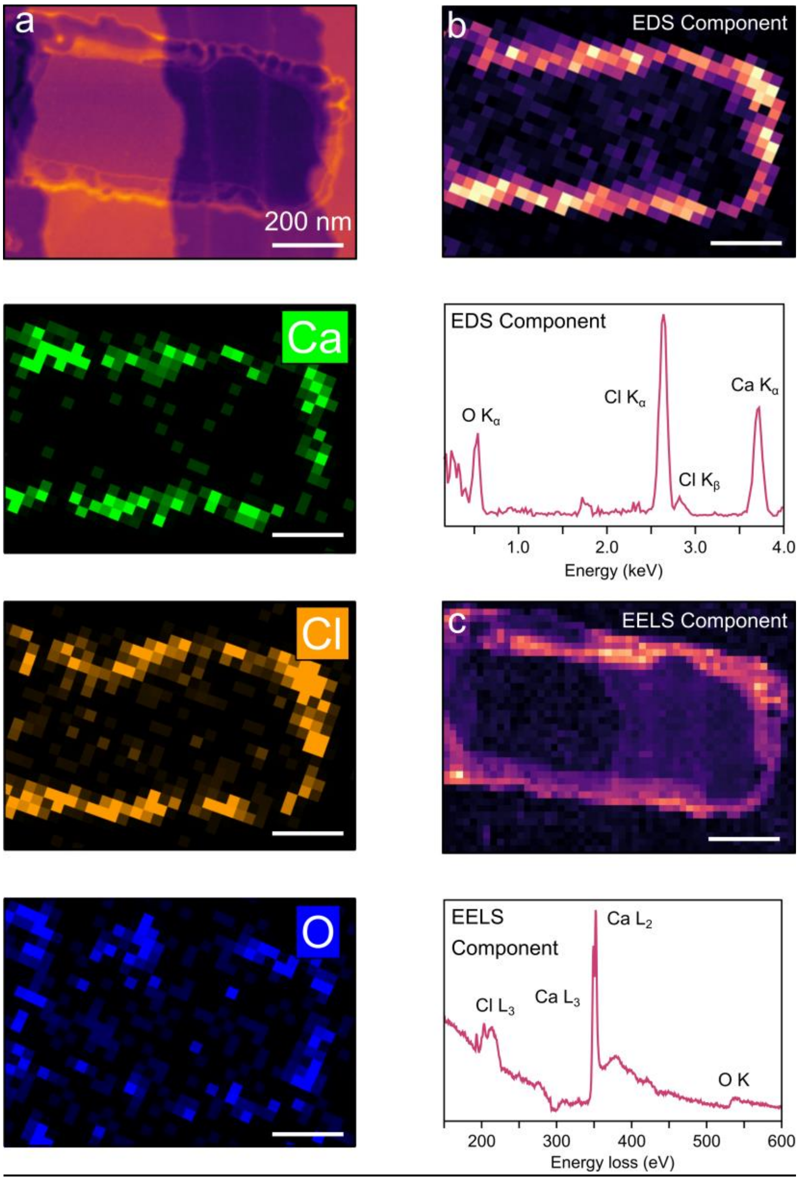

Figure S4.

(a) ADF-STEM micrograph of a horizontally positioned trench shaped well containing aqueous $CaCl_2$ solution prior to mixing alongside STEM-EDS elemental maps. The overlapping trough (aligned vertically in the image) contains aqueous Na_2CO_3 solution and is patterned in the bottom-hBN flake (close to beam exit). Decomposition of the b) EDS and c) EELS spectrum image yields a component showing co-localization of the Ca, Cl and O elements throughout the horizontally positioned trench area (corresponding to aqueous $CaCl_2$ solution). All scale bars are 200 nm.

Section 4: Beam-induced Nanofracture of Separation Membrane

The role of the MoS_2 separation membrane is to prevent premature mixing of the two encapsulated solutions until the 2D-MC is loaded into the microscope and the electron beam used to controllably initiate mixing. Both graphene and MoS_2 are susceptible to knock-on damage and radiolytic degradation when irradiated with a 200 kV electron beam, their respective knock-on thresholds being 80 keV and 90 keV respectively, though these are theoretical values and experimental measurements indicates the values may be lower in practice.[8,9] As such, the 200 kV electron beam we have used in our 2D-MC experiments induces knock-on damage at a similar rate in both materials and radiolytic effects will be less than those that occur with an 80 kV beam at similar fluence. Damage to the upper and lower graphene windows can cause liquid to leak into the TEM vacuum through nanopores, similar to that observed for earlier engineered GLCs (EGLCs), but it was found that by focusing the electron beam on the mono- or bilayer MoS_2 separation membrane, this was consistently damaged sooner than the thicker graphene windows. Suspended MoS_2 exhibits high elasticity and has a Young's modulus of 0.30 TPa,[10] however with sufficient loading, cracks form within the lattice and can propagate over micron-scale distances.

This degradation of MoS_2 under the electron beam has been well-characterized in recent years and we observe similar behavior in the suspended MoS_2 that separates the mixing cell compartments. We employ a similar strategy to that used by Ly et al.[11] to study cracking in MoS_2, where a high electron fluence is applied to a small (in this case 5 nm × 5 nm) area of MoS_2 in order to form a pore, from which cracks in the lattice propagate until the system stresses are sufficiently relaxed or the cracks are stabilised by adsorbents. Supplementary video SV1 shows dynamic cracking over a large scale, with individual atomic-resolution frames shown in Figure S5. A notable feature of these atomic-resolution STEM micrographs

is the smearing and splitting of FFT spots due to the MoS_2 either side of the being rotated as the crack tip widens as it propagates (see insets in Figure S5).

As the cracking phenomenon serves to achieve strain relaxation in the separation membrane, when the system is fully relaxed no further propagation of the cracks can be observed, meaning mixing products can be imaged on a stable support. In addition, we find that although these cracks are observed to occur over micrometer distances, they are pinned by the edge of the hBN well and do not extend beyond the cell boundaries. As a consequence, mixing can be induced in individual cells without compromising the integrity of the separation membrane in neighboring cells.

During the pore formation, it may be the case that C, S or Mo atoms are sputtered into the liquid cell itself, which may influence the reaction chemistry. However we expect this effect to be minimal, given the size of the irradiated area (equivalent to ~200-300 atoms) relative to the volume of the solution and the tendency for Mo to form stable metallic clusters at hole boundaries. Additionally there is no evidence to suggest a significant number of loose Mo or S atoms are drawn into the solution during cracking and the chemical reactions observed in this work are typically 30-150 nm from the initial pore.

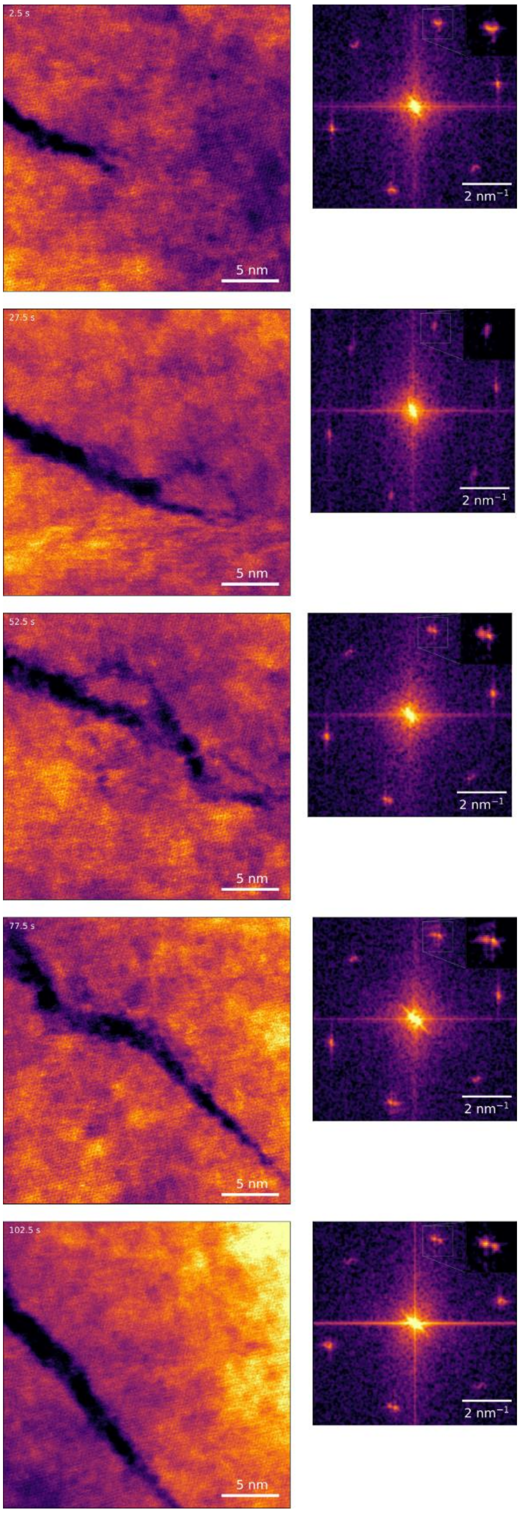

Figure S5.

Frames extracted from a video in SV1 that shows the propagation of a crack through the MoS_2 separation membrane due to the creation of a pore. Insets in the corner of the FFTs show the MoS_2 either side of the crack has rotated by an angle of 1-2° relative to each other.

Section 5: Image Processing

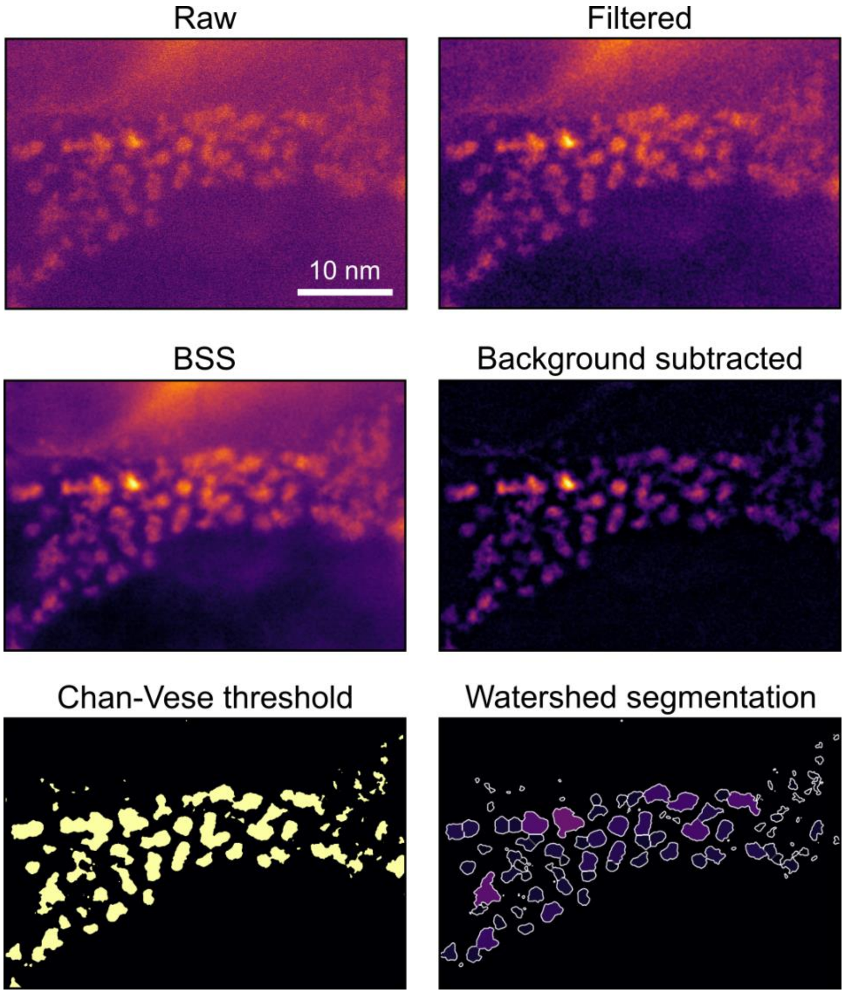

Figure S6.

A single frame from video SV3 at various stages in the image processing workflow. The raw image is first subject to a Weiner filter. Blind-source separation is then applied and a rolling-ball background subtraction method used to amplify particle signal to noise ratio. Chan-Vese thresholding is then applied to detect particle areas and a watershed transformation applied to segment individual particles, from which growth statistics can be extracted.

Section 6: Ion Activity Product upon Mixing Precursor Solutions

Assuming homogenous mixing of the 7.5 mM $CaCl_2$ and Na_2CO_3 solutions, the concentrations of calcium and carbonate in the mixed solution are each $3.75\cdot10^{-3}$ M. Given time for equilibration, we can calculate the total concentration of bound calcium carbonate due to ion association (ion pairs and pre nucleation clusters), $c_{eq.}$, since the free ion product is the relevant quantity for assessing the region of the phase diagram accessed after mixing. The ion association constant is on the order of 1500 M^{-1} such that: [12]

$$1500\ \mathrm{M}^{-1} = \frac{c_{eq}}{\left[3.75 \cdot 10^{-3}\mathrm{M} - c_{eq}\right]^2}$$

$$c_{eq} \approx 2.4 \cdot 10^{-3}\mathrm{M}$$

$$c(Ca^{2+}\ and\ CO_3^{2-}) \approx (3.75 - 2.4) \cdot 10^{-3}\mathrm{M} = 1.35 \cdot 10^{-3}\mathrm{M}$$

This allows calculation of the ion product after mixing, with ion association equilibration:

$$c(Ca^{2+})c(CO_3^{2-}) \approx 1.8 \cdot 10^{-6} M^2$$

According to the model developed for this reaction,[13] the experimentally verified ion activity product for the spinodal limit is:

$$IAP(spinodal) = \left[\frac{1}{1500}\right]^2 = 4.4 \cdot 10^{-7}$$

Thus, the ion product of calcium and carbonate for the system examined in this study is one order of magnitude higher than the calculated spinodal limit. Therefore, even when considering activity coefficients on the order of 0.7,[12] it is likely that the demonstrated 2D-MC system initially exists in the unstable regime of the phase diagram and spinodal decomposition will occur, producing dense liquid droplets. For incomplete or heterogeneous mixing, or insufficient time for the equilibration of ion association before decomposition, the initial IAP would be even higher than estimated here.

Section 7: Formation of Liquid Droplets upon Mixing of Precursor Solutions

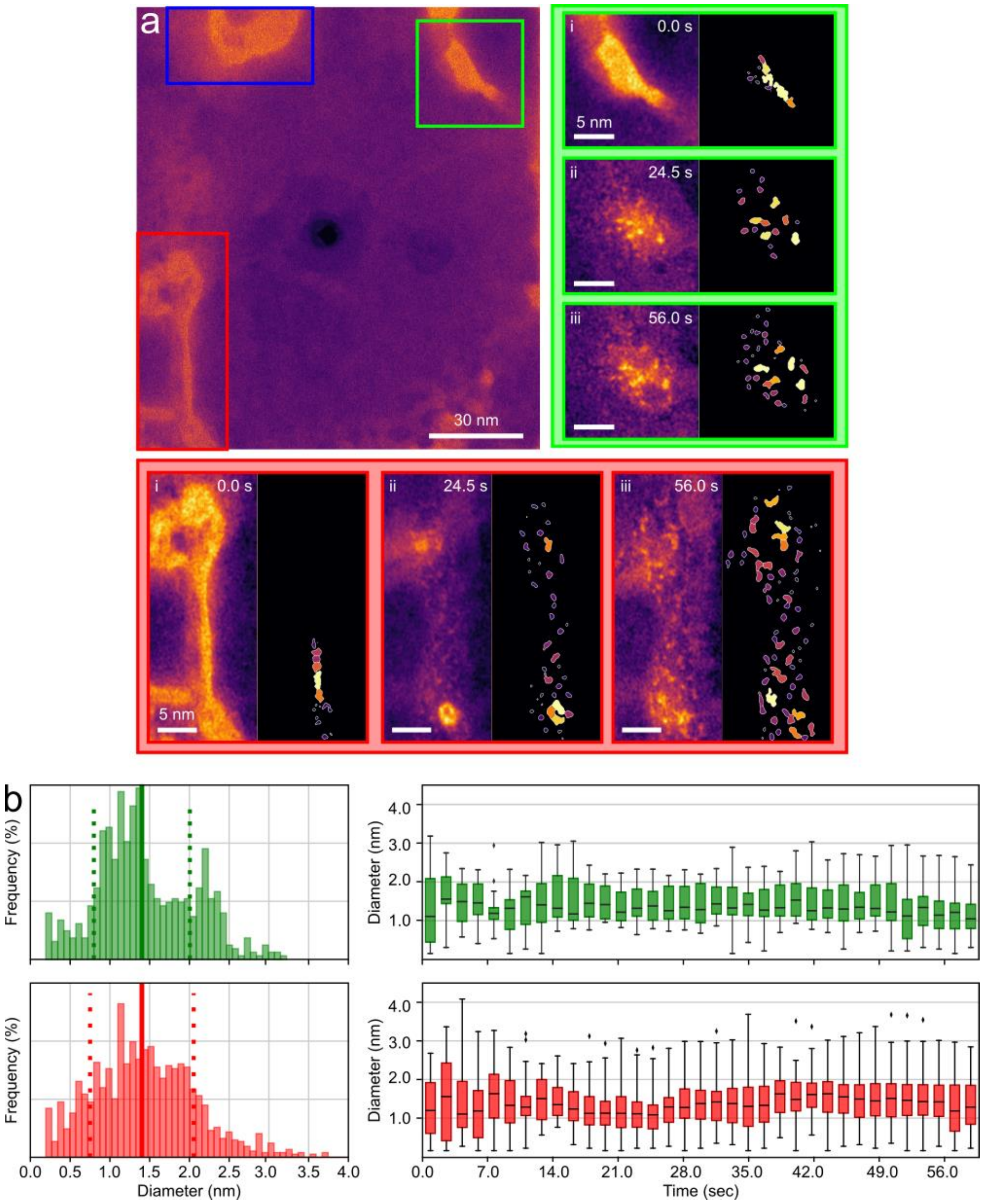

Figure S7.

(a) Additional series from the region (blue square) showcased in main figure 2 where high brightness liquid globules form near to a beam-induced pore. (b) Statistics acquired from the green and red regions in **a** reveal a size distribution with diameters from 0.6-2.5 nm and a slight decrease in size over time likely due to dehydration and beam-effects. Right side: histograms for size distribution over the entire timescale. The mean and first standard deviation are shown with solid and dotted lines respectively. Left side: box plots showing the distribution of size as a function of time, with the interquartile range displayed as a solid rectangle, the range by capped vertical lines and mean as a horizontal bar. Extreme outliers are displayed as singular points outside the range. Statistics from the early stage show large errors due to the difficulty of identifying particles within the globule.

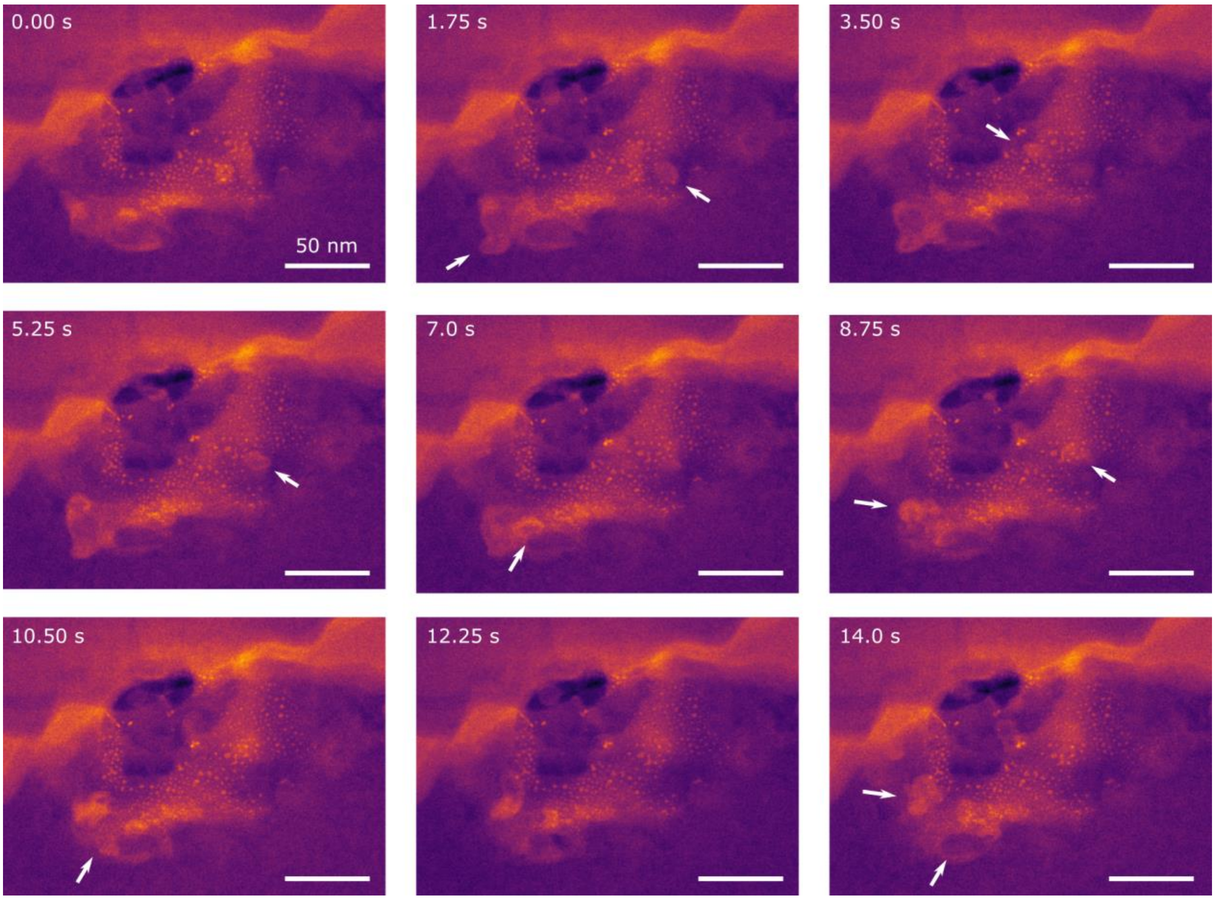

Figure S8.

Consecutive frames taken from SV4, which occurs after the particle formation in SV3, where highly mobile bright globules are located in the same region as the initial ionic nanodroplets formed outside the field of view of SV3. Arrows indicate some instances of the globules either changing morphologies or moving to new locations in the cell. These globules share characteristics with those observed in Figure 2 and so are considered spinodal decomposition products that continue to form as the solutions mix.

Section 8: Semi Quantitative EDS of Crystal Nucleated in 2D-MC

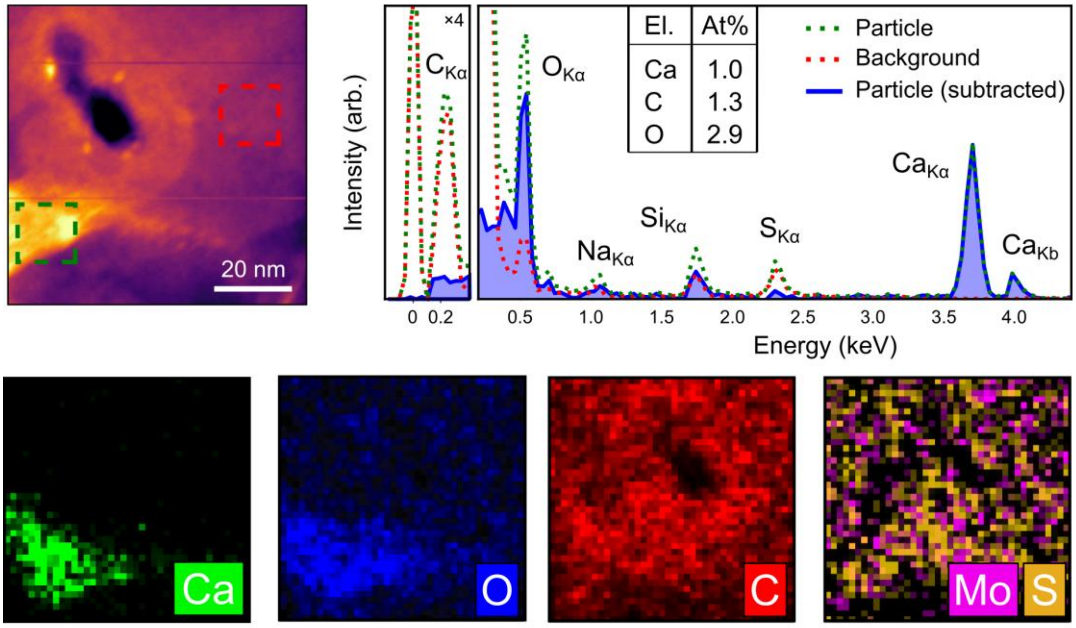

Figure S9.

STEM-EDS elemental analysis of the crystalline $CaCO_3$ particle from main text figure 4. A more accurate spectrum was computed by subtracting a background spectrum (integrated over the red square) from the particle spectrum (green square). Standardless Cliff-Lorimer analysis was performed to determine a ratio of ~ $CaCO_3$ (calculated atomic concentration is center inset in graph). Elemental maps in the lower panels show that the Ca signal is confined to the crystal area, O is distributed over the larger cell area (indicative of water), C, Mo and S are everywhere as expected.

Movie SV1.

Atomic resolution STEM imaging of crack propagation in the MoS_2 membrane instigated by the deliberate creation of a pore.

Movie SV2.

Evidence of mixing in 2D-MC where bright globules have formed around a hole in the MoS_2 separation membrane. The globules are observed to retract under electron beam illumination and form smaller nanodroplets.

Movie SV3.

Formation of amorphous calcium carbonate from phase-separated nanodroplet species as a consequence of mixing precursor solutions. Statistics relating to the species size and morphology were extracted from the video using segmentation and labelling, with the results presented in main text Figure 3.

Movie SV4.

Lower magnification ADF-STEM video of the area examined in Figure 3 and Movie SV3. Bright globules can be observed moving over the mixing area where particles have nucleated outside the original high magnification imaging area.

References

[1] T. Malis, S. C. Cheng, R. F. Egerton, *J. Electron Microsc. Tech.* **1988**, *8*, 193.

[2] K. L. Jungjohann, J. E. Evans, J. A. Aguiar, I. Arslan, N. D. Browning, *Microsc.*

Microanal. **2012**, *18*, 621.

[3] M. N. Yesibolati, S. Laganá, S. Kadkhodazadeh, E. K. Mikkelsen, H. Sun, T. Kasama, O. Hansen, N. Zaluzec, K. Mølhave, *Nanoscale* **2020**, DOI 10.1039/D0NR04352D.

[4] J. M. Yuk, J. Park, P. Ercius, K. Kim, D. J. Hellebusch, M. F. Crommie, J. Y. Lee, A. Zettl, A. P. Alivisatos, *Science* **2012**, *335*, 61.

[5] N. Noh, J. Park, J. S. Park, K. Koo, J. Y. Park, J. M. Yuk, *Lab Chip* **2020**, *20*, 2796.

[6] J. S. Bunch, S. S. Verbridge, J. S. Alden, A. M. Van Der Zande, J. M. Parpia, H. G. Craighead, P. L. McEuen, *Nano Lett.* **2008**, *8*, 2458.

[7] E. A. Lewis, S. J. Haigh, T. J. A. Slater, Z. He, M. A. Kulzick, M. G. Burke, N. J. Zaluzec, *Chem. Commun.* **2014**, *50*, 10019.

[8] H.-P. P. Komsa, J. Kotakoski, S. Kurasch, O. Lehtinen, U. Kaiser, A. V. Krasheninnikov, *Phys. Rev. Lett.* **2012**, *109*, 035503.

[9] T. Lehnert, O. Lehtinen, G. Algara-Siller, U. Kaiser, *Appl. Phys. Lett.* **2017**, *110*, 033106.

[10] A. Castellanos-Gomez, M. Poot, G. A. Steele, H. S. J. van der Zant, N. Agraït, G. Rubio-Bollinger, *Nanoscale Res. Lett.* **2012**, *7*, 1.

[11] T. H. Ly, J. Zhao, M. O. Cichocka, L. J. Li, Y. H. Lee, *Nat. Commun.* **2017**, *8*, 14116.

[12] M. Kellermeier, A. Picker, A. Kempter, H. Cölfen, D. Gebauer, *Adv. Mater.* **2014**, *26*, 752.

[13] J. T. Avaro, S. L. P. Wolf, K. Hauser, D. Gebauer, *Angew. Chemie - Int. Ed.* **2020**, *59*, 6155.
